# Thymoquinone Potentiates the Effect of Phenytoin against Electroshock-Induced Convulsions in Rats by Reducing the Hyperactivation of m-TOR Pathway and Neuroinflammation: Evidence from In Vivo, In Vitro and Computational Studies

**DOI:** 10.3390/ph14111132

**Published:** 2021-11-08

**Authors:** Faheem Hyder Pottoo, Mohammed Salahuddin, Firdos Alam Khan, Fadhel Alomar, Marwa Abdullah AL Dhamen, Abrar Fouad Alhashim, Hawra Hussain Alqattan, Mohamed S. Gomaa, Mohammad N. Alomary

**Affiliations:** 1 Department of Pharmacology, College of Clinical Pharmacy, Imam Abdulrahman Bin Faisal University, P.O. Box 1982, Dammam 31441, Saudi Arabia; falomar@iau.edu.sa (F.A.); 2160005543@iau.edu.sa (M.A.A.D.); 2150006408@iau.edu.sa (A.F.A.); 2160002828@iau.edu.sa (H.H.A.); 2 Department of Clinical Pharmacy Research, Institute for Research and Medical Consultations, Imam Abdulrahman Bin Faisal University, P.O. Box 1982, Dammam 31441, Saudi Arabia; msalahuddin@iau.edu.sa; 3 Department of Stem Cell Research, Institute for Research and Medical Consultations, Imam Abdulrahman Bin Faisal University, P.O. Box 1982, Dammam 31441, Saudi Arabia; fakhan@iau.edu.sa; 4 Department of Pharmaceutical Chemistry, College of Clinical Pharmacy, Imam Abdulrahman Bin Faisal University, P.O. Box 1982, Dammam 31441, Saudi Arabia; msmmansour@iau.edu.sa; 5 National Centre for Biotechnology, Kind Abdulaziz City for Science and Technology (KACST), P.O. Box 1982, Riyadh 11442, Saudi Arabia

**Keywords:** phenytoin, thymoquinone, PI3K/Akt/m-TOR signaling, grand mal seizures, neuronal inflammation, convulsions, molecular docking, neuroprotection, cooperative binding

## Abstract

Epilepsy is a chronic neurodegenerative disease characterized by multiple seizures, hereto 35% of patients remain poor responders. Phenytoin (PHT; 20 and 40 mg/kg) and thymoquinone (THQ; 40 and 80 mg/kg) were given alone and as a low dose combination for 14 days (p.o), prior to challenge with maximal electroshock (MES; 180 mA, 220 V, 0.2 s). Apart from observing convulsions, hippocampal mTOR, IL-1β, IL-6 and TNF-α levels were measured. Hippocampal histomorphological analysis was also conducted. In vitro cell line studies and molecular docking studies were run in parallel. The results revealed the synergistic potential of the novel duo-drug combination regimen: PHT (20 mg/kg) and THQ (40 mg/kg) against MES-induced convulsions. MES amplified signaling through mTOR, and inflated the levels of proinflammatory markers (IL-1β, IL-6 and TNF-α), which was significantly averted (*p* < 0.001) with the said drug combination. The computational studies revealed that PHT and THQ cooperatively bind the active site on Akt (upstream target of m-TOR) and establish a good network of intermolecular interactions, which indicates the sequential inhibition of PI3K/Akt/m-TOR signaling with the combination. The combination also increased cell viability by 242.81% compared to 85.66% viability from the the toxic control. The results suggest that the PHT and THQ in combination possesses excellent anticonvulsant and neuroprotective effects.

## 1. Introduction

Epilepsy is a chronic neuropathological state affecting the brain resulting from excessive neuronal activity and periodical incidents of seizures [1,2]. The international league against epilepsy (ILAE) described epileptic seizure as “transient occurrence of signs and symptoms due to abnormal excessive or synchronous neuronal activity in the brain” [3]. A recent study considered epilepsy one of the most common neurological disorders, besides stroke and Alzheimer’s disease. Approximately, 70 million people globally have epilepsy, affecting people of all ages with a variety of possible causes and presentations [4]. The range of estimated incidences of epilepsy have fallen over the last years in high economic countries compared to low economic countries [5]. Several mechanisms lead to seizure generation in a healthy brain which include dysregulation between excitatory (glutamatergic) and inhibitory (GABAergic) signaling [6]. However, the pathophysiology of epileptic seizure is diverse depending on the type of seizure [7].

Phenytoin (PHT) has an anticonvulsant effect against a variety of seizure disorders. It acts by blockage of membrane sodium channels which are responsible for the action potential (23). Although PHT is widely used in epilepsy treatment, it has many undesirable effects that diminish its usage in clinical practice. It is characterized by a narrow therapeutic index which leads to intoxication (24). Thymoquinone (THQ) is the major ingredient of *Nigella sativa*, recounted in the literature as a neuroprotective agent. THQ possesses antinociceptive effects by activation of κ-opioid receptor subtypes, which in turn leads to an anticonvulsant effect. THQ prolonged the latency of myoclonic seizure and reduced its duration (26).

mTOR is a protein kinase from the serine/threonine family, an essential protein in controlling various anabolic process such as cell metabolism, growth, proliferation, and synthesis of proteins and lipids [8,9]. The mTOR activity is regulated by PI3K/Akt [10]. mTOR exists in two forms: mTORC1 and mTORC2. In the nervous system, mTOR regulates synaptic transmission, plasticity, neurogenesis, morphology and neural network activity [11,12]. It is reasonable that mTOR dysregulation is linked to several neurological disorders [13]. Tuberous sclerosis complex (TSC) is a hereditary condition related to the mTOR pathway, which is a benign tumor that affects several organs, including the heart, brain, lungs, skin, eyes and kidneys [14,15]. Mutations in the *TSC1* or *TSC2* genes cause deficiency in normal inhibition of mTORC1, which leads to disruptions in synaptogenesis, neuronal hyperactivity and hypertrophy [16,17,18]. Consequently, these abnormalities affect the seizure threshold by changing neuronal connectivity, cortical morphology at the systems level and neuronal hyperexcitability at the cellular level [19]. The PI3K/Akt/mTOR might exhibit cooperative binding with ligands (PHT/THQ). The cooperative binding is homotropic when ligand impacts the binding of similar ligands, or else heterotropic when ligand effects binding of dissimilar ligands [20]. The cooperativity could be positive or negative depending on whether the binding of a ligand molecule escalates or reduces the chance of binding another ligand molecule [20].

Many epileptic patients present inflammatory responses in clinical presentation, characterized by elevation in the body temperature, C-reactive protein, changes in other vital signs (SaO_2_, HR, RR, SBP, DBP etc.) [21], as well as perinatal injuries such as hypoxic ischemic encephalopathy. The inflammatory response results from extensive release of proinflammatory mediators, which contribute to neuronal damage and lead to seizure exacerbation [22,23]. The IL-1β, a pro-inflammatory cytokine located in activated astrocytes and microglia, plays a critical role in the induction of seizures through increasing neuronal hyperexcitability, stimulating glutamate release from astrocytes, and inhibiting glutamate reuptake. Increased levels of IL-1β weaken physiologic synaptic elasticity and damage the neurons. Interleukin-6 (IL-6) is a cytokine responsible in the regulation of immune and inflammatory responses. Several clinical studies indicated that IL-6 levels are elevated in the blood serum and cerebrospinal fluid (CSF) in patients with neurodegenerative disease or brain injury. Other studies reveal that IL-6 in itself is enough to induce epileptic activity either ex vivo or in vivo. Tumor necrosis factor (TNF-α) is a pro-inflammatory cytokine which regulates glutamate receptor trafficking. TNF receptor 1 (TNFR1) and TNF receptor 2 (TNFR2) escalate synaptic glutamate levels and excite neurons. A positive correlation was reported between TNF-α and epileptogenesis in a kindling epilepsy model [24].

Over the last decades, people have been suffering from the neurological complications of epilepsy, as well as severe toxic effects from typical pharmacological treatments [25,26]. As a result, none of the current approaches have been implemented to completely control the epileptic seizures. The reason for this is the lack of current AEDs to intercept the neurodegenerative cascades in an efficient manner, as the neurodegeneration in the process of epileptogenesis changes the receptor properties, which reduces the binding of AEDs with their receptors and thereby decreases the effectiveness of AEDs. Thus, we sought to combine PHT (anticonvulsant) with THQ (neuroprotective), so as to prevent the neurodegenerative changes and enhance the clinical efficacy (that at low doses would further reduce the adverse effects).

## 2. Results

### 2.1. Impact of Phenytoin (PHT), Thymoquinone (THQ) Alone and in Combination Therapy against Electroshock-Induced THLE

**Figure 1:**
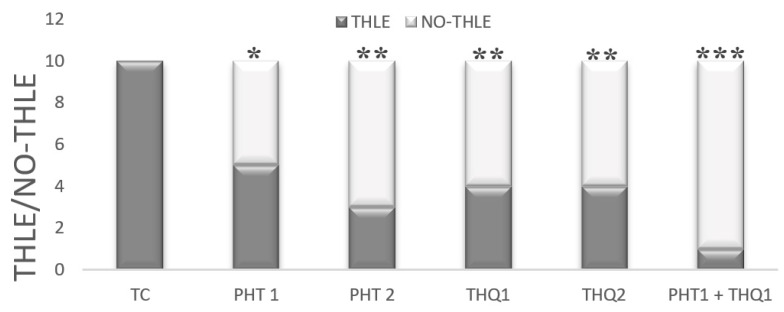
Effect of PHT (20 and 40 mg/kg), THQ (40 and 80 mg/kg) and PHT (20 mg/kg) + THQ (40 mg/kg) against MES-produced THLE in rats. Fischer’s exact test (one tailed) was used to compare the ratio of THLE/NO THLE. All groups were compared with the TC group. PHT, THQ, TC, MES and THLE indicate phenytoin, thymoquinone, toxic control, maximal electric shock and tonic hind limb extension, respectively. * *p* < 0.01, ** *p* < 0.05, *** *p* < 0.001.

All rats in the toxic control group challenged with MES (180 mA, 220 V for 0.20 s) developed THLE (i.e., THLE/NO-THLE = 10/0). Pretreatment with PHT at 20 mg/kg and 40 mg/kg for 14 days reduced (*p* < 0.05, *p* < 0.01) the number of rats exhibiting electroshock-induced THLE (in a dose dependent manner). Pretreatment with THQ at 40 and 80 mg/kg also reduced (*p* < 0.01) the number of rats exhibiting THLE (similar efficacy at both doses). Interestingly, pretreatment with low dose combination of PHT (20 mg/kg) and THQ (40 mg/kg) for 14 days displayed the highest reduction (*p* < 0.001) in the number of rats exhibiting THLE (i.e., THLE/NO-THLE = 1/9). It seems that the low dose combination of PHT (20 mg/kg) and THQ (40 mg/kg) exhibited synergism to raise the threshold against electroshock-provoked THLE, thus it appears to be a judicious approach in the treatment of generalized seizures (Figure 1).

### 2.2. Impact of Phenytoin (PHT), Thymoquinone (THQ) Alone and in Combination Therapy on Recovery Time Post Electroshock-Induced Convulsions

**Figure 2:**
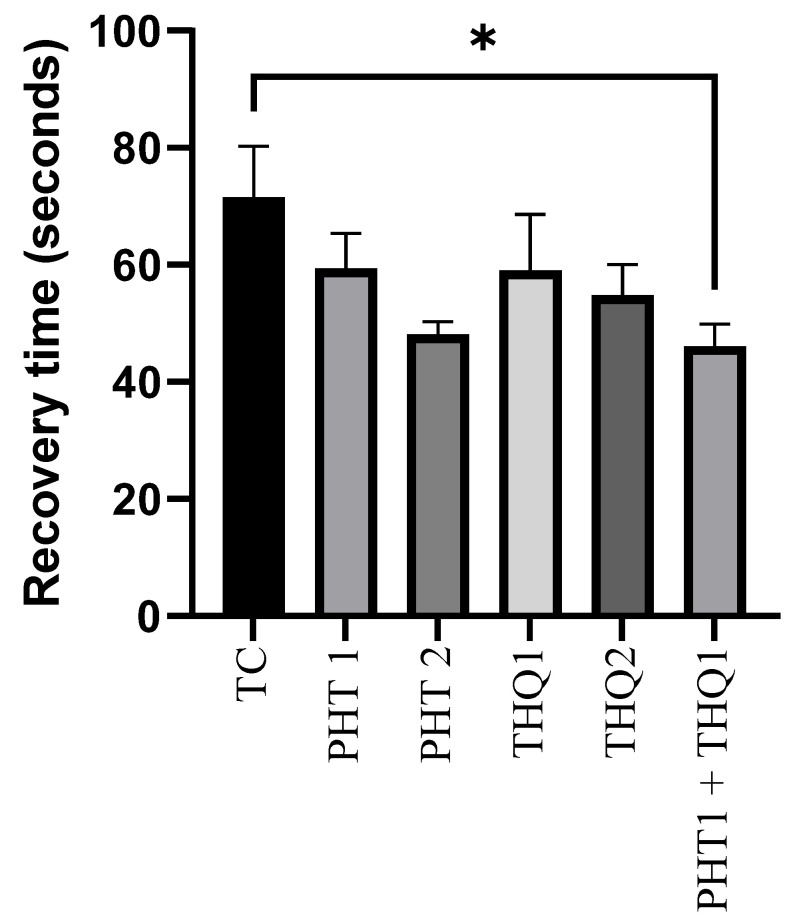
Effect of PHT (20 and 40 mg/kg), THQ (40 and 80 mg/kg) and PHT (20 mg/kg) + THQ (40 mg/kg) on recovery time following MES-induced convulsions in rats. One-way ANOVA followed by Dunnet’s test was used to compare quantitative variables from the toxic control vs. other groups. PHT, THQ, TC and MES indicate phenytoin, thymoquinone, toxic control and maximal electric shock, respectively. * *p* < 0.05.

The recovery after MES (180 mA, 220 V for 0.20 s)-induced convulsions was improved with PHT (20 and 40 mg/kg) and THQ (40 and 80 mg/kg), compared with the toxic control group. However, the significant (*p* < 0.05) reduction in recovery time was observed only with the low dose combinatorial therapy of PHT (20 mg/kg) and THQ (40 mg/kg) (46.12 s vs. 71.5 s from the toxic control group). These results indicate that the current propitious drug combination counteracts electrical insult via diverse pathways (Figure 2).

### 2.3. Effect of Phenytoin (PHT), Thymoquinone (THQ) Alone and in Combination Therapy on Hippocampal mTOR Levels

**Figure 3:**
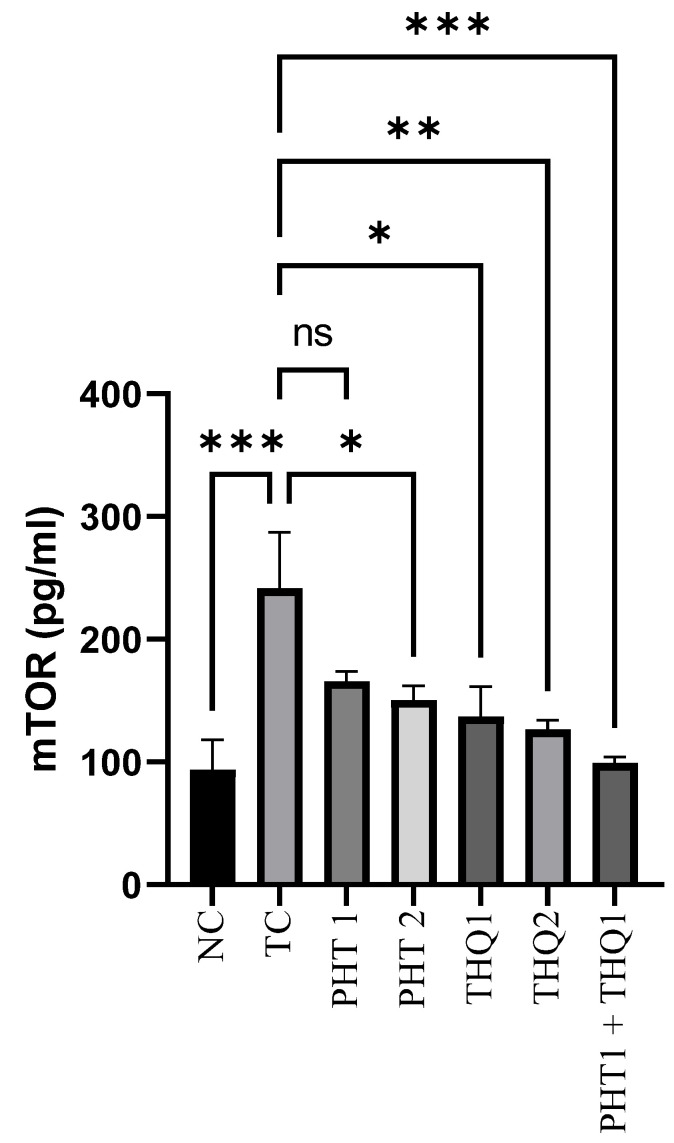
Effect of PHT (20 and 40 mg/kg), THQ (40 and 80 mg/kg) and PHT (20 mg/kg) + THQ (40 mg/kg) on hippocampal mTOR levels following MES-induced convulsions in rats. One-way ANOVA followed by Dunnet’s test was used to compare quantitative variables from the toxic control vs. other groups. PHT, THQ, TC and MES indicate phenytoin, thymoquinone, toxic control and maximal electric shock, respectively. * *p* < 0.05, ** *p* < 0.01, *** *p* < 0.001.

MES (180 mA, 220 V for 0.20 s) produced a substantial rise (*p* < 0.001) in hippocampal mTOR levels in the toxic control group, compared to normal control. PHT (40 mg/kg) and THQ (40 and 80 mg/kg) reduced (*p* < 0.05, *p* < 0.05, *p* < 0.01) mTOR levels, respectively. However, the most marked reduction (*p* < 0.001) of m-TOR levels was exhibited by the low dose combination regimen of PHT (20 mg/kg) and THQ (40 mg/kg). All treated groups were compared with toxic control group. The results indicate hyperactivation of the m-TOR pathway in epileptic states, resulting in hyperexcitability and epileptogenesis, which seem to be restricted by the synergistic combination of PHT and THQ (at low doses) (Figure 3).

### 2.4. Effect of Phenytoin (PHT), Thymoquinone (THQ) Alone and in Combination Therapy on Inflammatory Markers in Hippocampus

#### 2.4.1. Effect on Hippocampal IL-1β Levels

**Figure 4:**
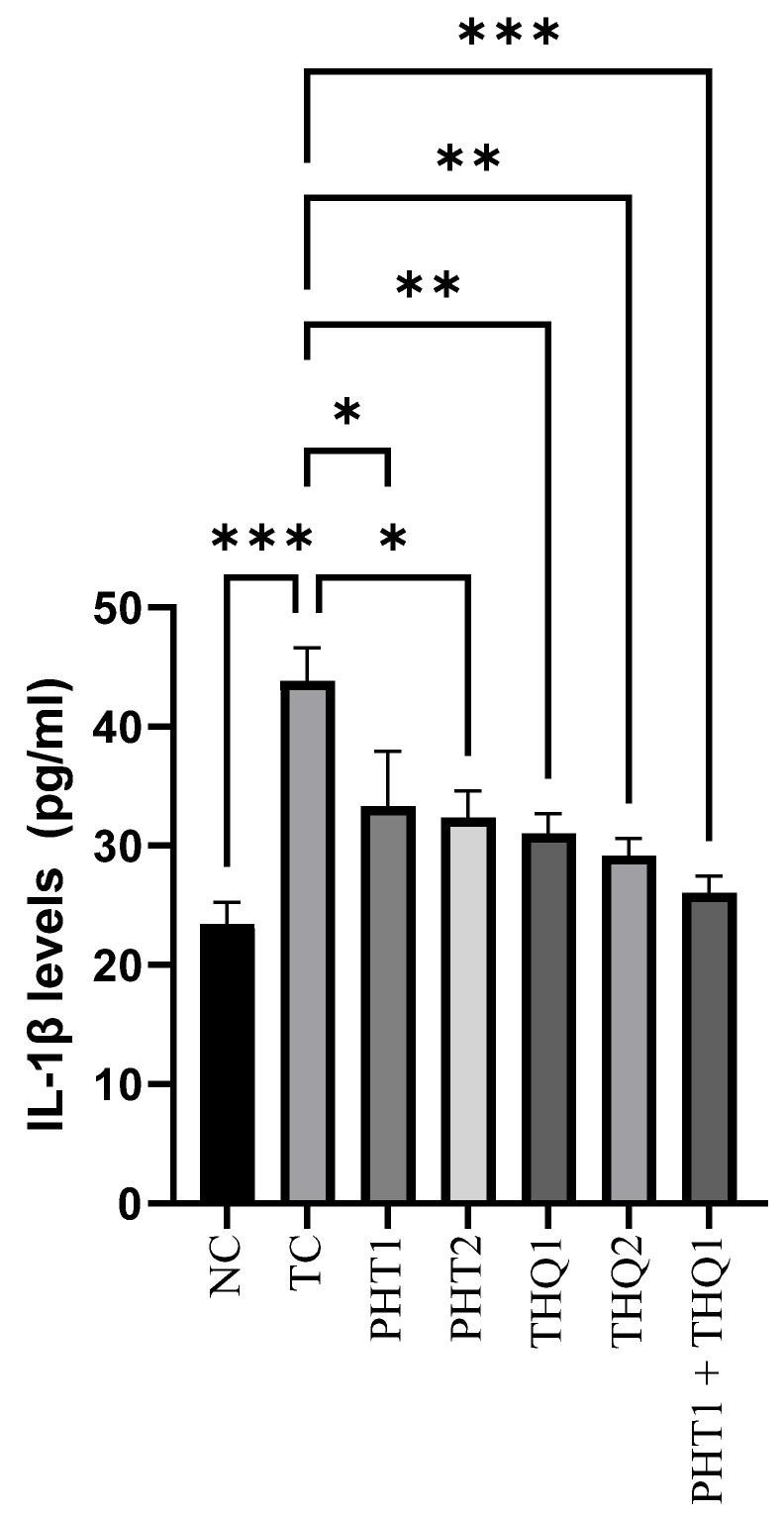
Effect of PHT (20 and 40 mg/kg), THQ (40 and 80 mg/kg) and PHT (20 mg/kg) + THQ (40 mg/kg) on hippocampal *IL*-1β levels following MES-induced convulsions in rats. One-way ANOVA followed by Dunnet’s test was used to compare quantitative variables from the toxic control vs. other groups. PHT, THQ, TC and MES indicate phenytoin, thymoquinone, toxic control and maximal electric shock, respectively. * *p* < 0.05, ** *p* < 0.01, *** *p* < 0.001.

MES (180 mA, 220 V for 0.20 s) induced a substantial rise (*p* < 0.001) in hippocampal IL-1β levels in the toxic control group, compared to the normal control. PHT (20 and 40 mg/kg) reduced (*p* < 0.05) IL-1β levels. THQ (40 & 80 mg/kg) also reduced (*p* < 0.01) IL-1β levels. However, the most substantial reduction (*p* < 0.001) was exhibited by the low dose combination regimen of PHT (20 mg/kg) and THQ (40 mg/kg). All treated groups were compared with the toxic control group. The results indicate electroshock-induced brain inflammation, resulting in hyperactivation and neuroinflammation, which were counteracted well with the synergistic combination of PHT and THQ (at low doses) (Figure 4).

#### 2.4.2. Effect on Hippocampal *IL*-6 Levels

**Figure 5:**
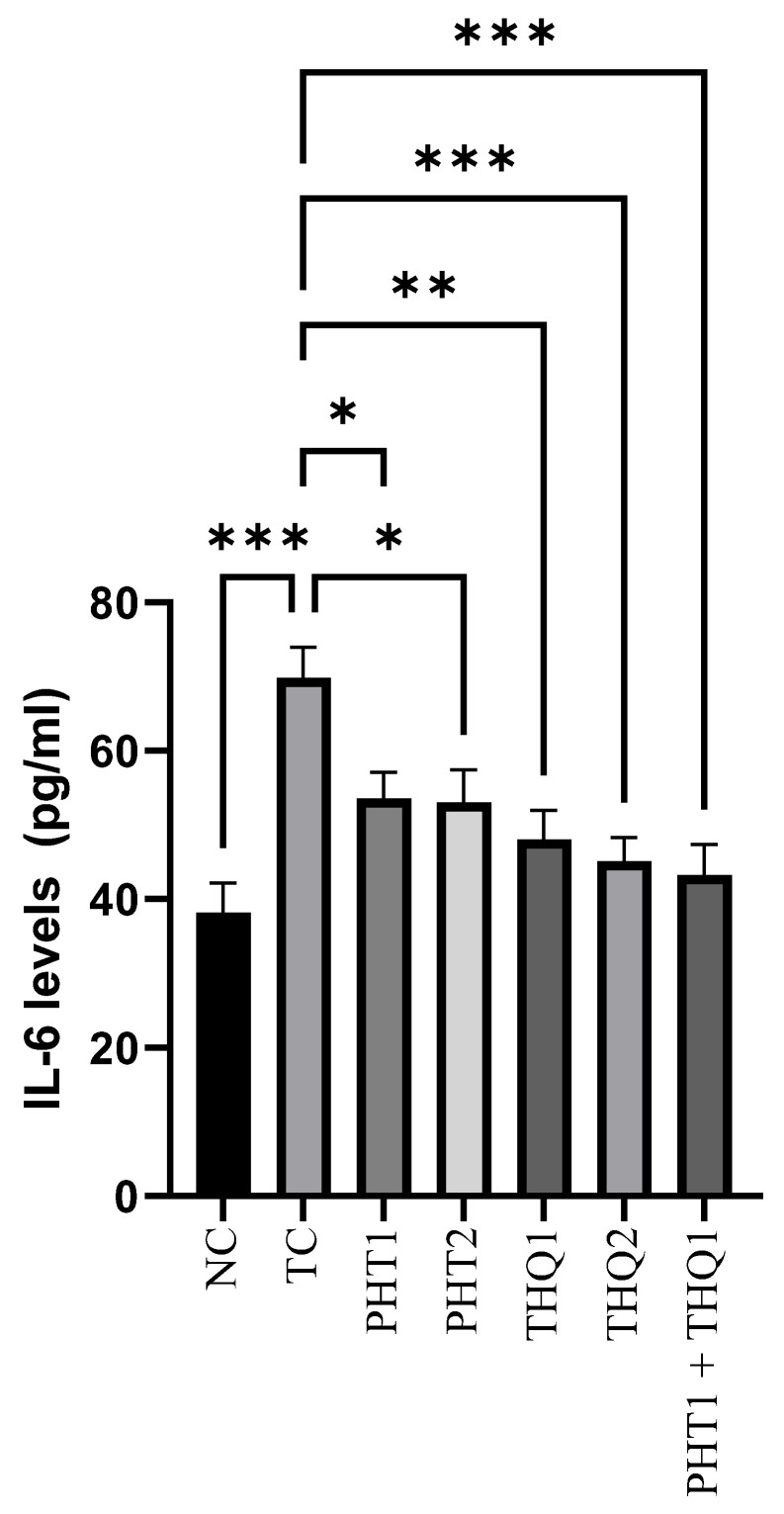
Effect of PHT (20 and 40 mg/kg), THQ (40 and 80 mg/kg) and PHT (20 mg/kg) + THQ (40 mg/kg) on hippocampal *IL*-6 levels following MES-induced convulsions in rats. One-way ANOVA followed by Dunnet’s test was used to compare quantitative variables from the toxic control vs. other groups. PHT, THQ, TC and MES indicate phenytoin, thymoquinone, toxic control and maximal electric shock, respectively. * *p* < 0.05, ** *p* < 0.01, *** *p* < 0.001.

MES (180 mA, 220 V for 0.20 s) produced a substantial rise (*p* < 0.001) in hippocampal *IL*-6 levels in the toxic control group, compared to the normal control. PHT (20 and 40 mg/kg) reduced (*p* < 0.05) *IL*-6 levels. THQ (40 and 80 mg/kg) also reduced (*p* < 0.01, *p* < 0.001) *IL*-6 levels in a dose dependent manner. However, the low dose combination regimen of PHT (20 mg/kg) and THQ (40 mg/kg) caused the greatest reduction (*p* < 0.001) in *IL*-1β levels, which indicates amelioration of brain inflammation with the low dose drug combination. All treated groups were compared with the toxic control group (Figure 5).

#### 2.4.3. Effect on Hippocampal TNF-α Levels

**Figure 6:**
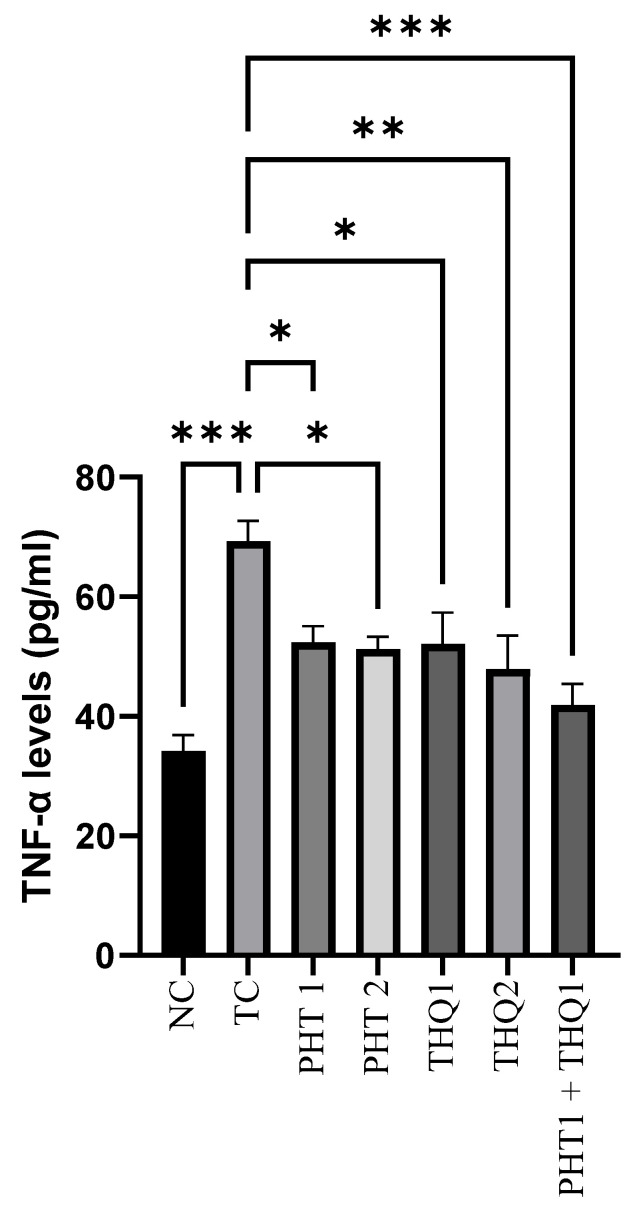
Effect of PHT (20 and 40 mg/kg), THQ (40 and 80 mg/kg) and PHT (20 mg/kg) + THQ (40 mg/kg) on hippocampal TNF-α levels following MES-induced convulsions in rats. One-way ANOVA followed by Dunnet’s test was used to compare quantitative variables from the toxic control vs. other groups. PHT, THQ, TC and MES indicate phenytoin, thymoquinone, toxic control and maximal electric shock, respectively. * *p* < 0.05, ** *p* < 0.01, *** *p* < 0.001.

MES (180 mA, 220 V for 0.20 s) induced a substantial increase (*p* < 0.001) in hippocampal TNF-α levels in the toxic control group, compared to the normal control. PHT (20 & 40 mg/kg) reduced (*p* < 0.05) TNF-α levels. THQ (40 & 80 mg/kg) also reduced (*p* < 0.05, *p* < 0.01) TNF-α levels in a dose dependent manner. However, the most significant reduction (*p* < 0.001) was exhibited by the low dose combination regimen of PHT (20 mg/kg) and THQ (40 mg/kg). The results indicate that electroshock-induced brain inflammation results in hyperactivation and neurodegeneration, which was counteracted well with the synergistic combination of PHT and THQ (at low doses). All treated groups were compared with the toxic control group (Figure 6).

### 2.5. Effect of Phenytoin (PHT), Thymoquinone (THQ) Alone and in Combination Therapy on Hippocampal Neuronal Damage

**Figure 7:**
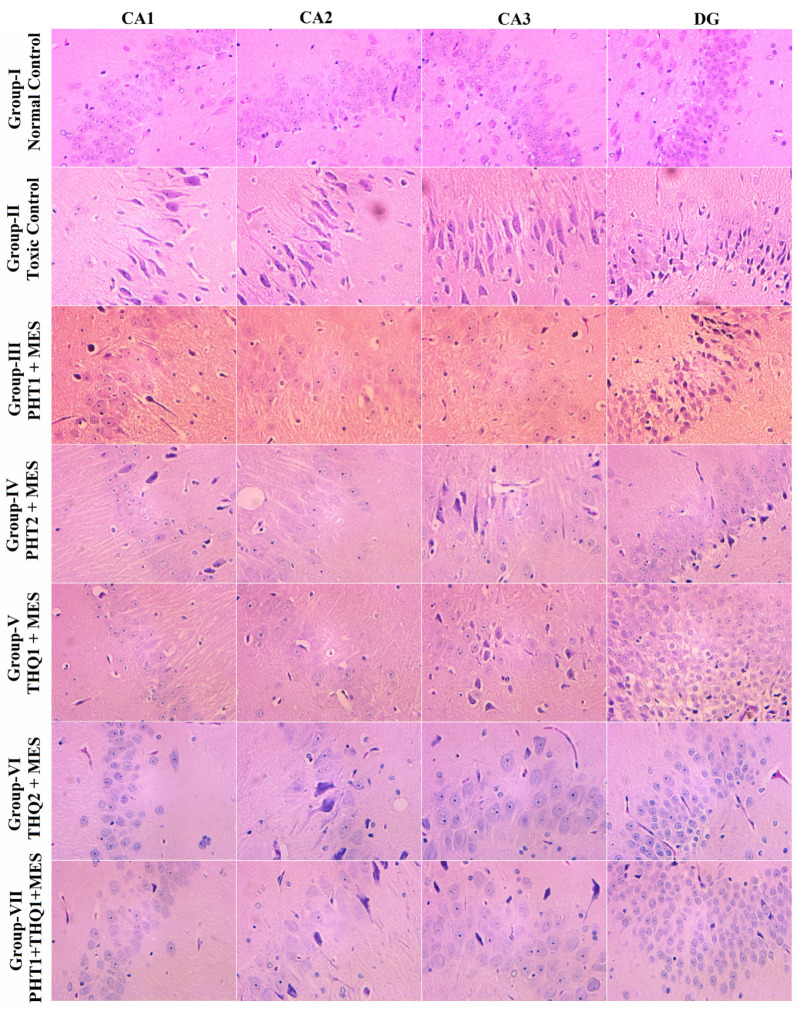
Illustrative photomicrographs depicting H/E-stained rat hippocampal sections revealing the effect of PHT (20 and 40 mg/kg), THQ (40 and 80 mg/kg), and PHT (20 mg/kg) + THQ (40 mg/kg) against electroshock-produced neuronal degeneration. MES (180 mA, 220 V for 0.20 s) reduced neuronal density in all hippocampal regions—CA1, CA2, CA3 and DG—and induced hefty pyknosis in the DG region (Group II). PHT (20 and 40 mg/kg) dose dependently resisted pyknosis, but neuronal loss is evident in all regions (Group III–IV). THQ (40 and 80 mg/kg) also resisted electroshock-induced neuronal damage (Group V–VI) in a dose dependent manner. The low dose synergistic regimen of PHT (20 mg/kg) and THQ (40 mg/kg) exhibited excellent neuroprotectant properties as evidenced by largely protected neuronal morphology and relatively high neuronal density with meagre pyknosis (Group-VII).

In the toxic control group, MES (180 mA, 220 V for 0.20 s) delivered via auricular electrodes culminated in a hefty neuronal loss in all hippocampal regions, CA1, CA2, CA3 and DG, In addition, DG revealed severe pyknosis. Monotherapy with THQ (20 and 40 mg/kg) was more effective in restricting neuronal damage and pyknosis than that of PHT (20 and 40 mg/kg). However, the low dose combination regimen of PHT (20 mg/kg) and THQ (40 mg/kg) revealed substantial neuroprotection by preserving the neuronal density and morphology, and restricting pyknosis in all hippocampal subregions (Figure 7).

### 2.6. Effect of Phenytoin (PHT), Thymoquinone (THQ) Alone and in Combination Therapy on PTZ-Induced Cellular Degeneration on HEK-293 Cells

**Figure 8:**
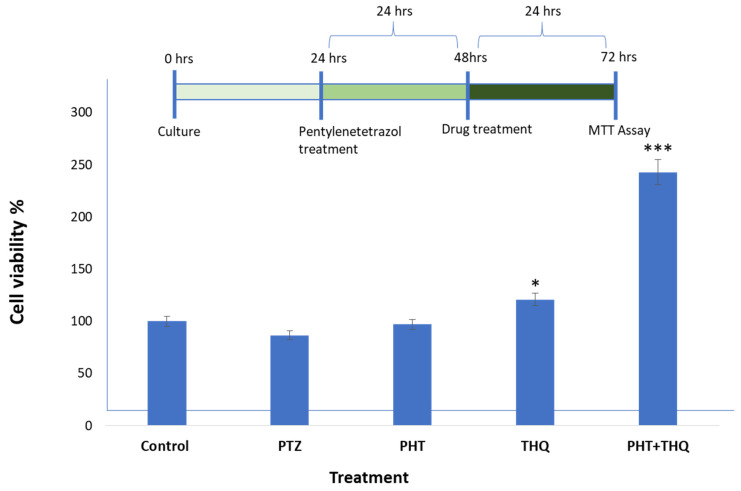
The complete cycle of treatment and the cell viability assay by MTT assay: control group (no drug treatment), PTZ (0.6 µg/mL), PHT (0.33 µg/mL), THQ (0.33 µg/mL), PHT + THQ (0.33 µg/mL + 0.66 µg/mL) * *p* < 0.01, *** *p* < 0.0001. PHT, THQ and PTZ indicate phenytoin, thymoquinone and pentylenetetrazol, respectively.

Cell viability assay: We studied cell viability by MTT assay. After 24 h of PTZ (0.6 µg/mL) treatment, we checked the cell viability which was reduced to 85.66% compared to the control (100%). In order to evaluate the impact of the drugs PHT (0.33 µg/mL) and THQ (0.33 µg/mL) on the PTZ-treated cells, we tested the cell viability after 24 h. The cells treated with PHT (0.33 µg/mL) and THQ (0.33 µg/mL) alone showed an increase in the cell viability to 97.03% and 120.74%, respectively. However, for the cells treated with the combination regimen (PHT + THQ) in the ratio of 1:2, the cell viability escalated to 242.81%. All treated groups were compared with the toxic control, i.e., PTZ-treated cells (Figure 8).

### 2.7. Molecular Docking

**Figure 9:**
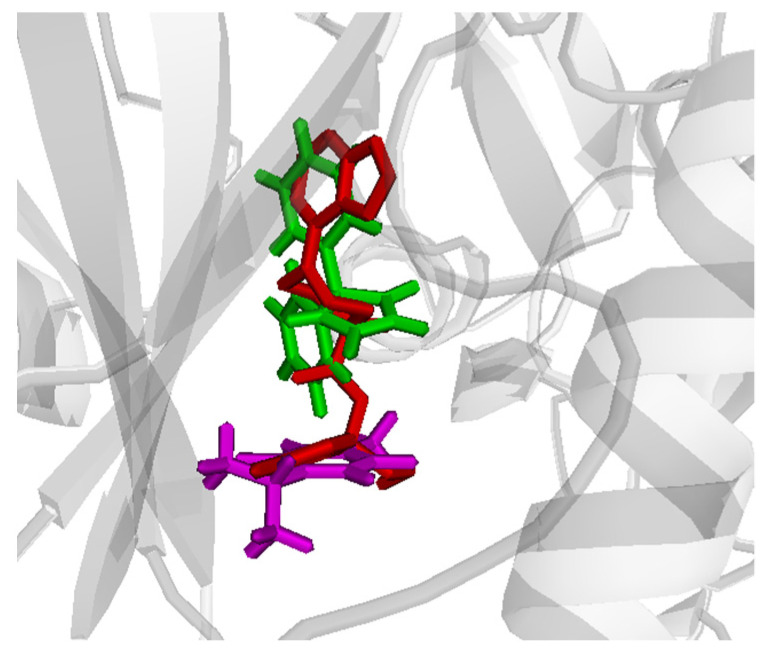
Comparative binding positions of co-bound PHT (green) and THQ (magenta) with the reference crystallized ligand (red) bound to the Akt active site. PHT and THQ indicate phenytoin and thymoquinone.

PHT and THQ were tested through molecular docking simulation for their co-binding ability to the Akt and PI3K active sites. Ligands were docked sequentially and in different orders in the target active sites and the binding scores were calculated for each single ligand and for the co-bound complex. Comparative docking was also performed through comparing the ligand positions in the co-bound form with the reference crystallized inhibitor. The docking scores are shown in Table 1. There are slightly better affinities for Akt in the case of single compounds, and the co-bound complex. The best results were obtained in case of docking PHT first (Table 1). However, interesting binding poses were noted for Akt, where the co-bound drugs almost superpose the reference crystallized ligand in the active site (Figure 9).

**Figure 10:**
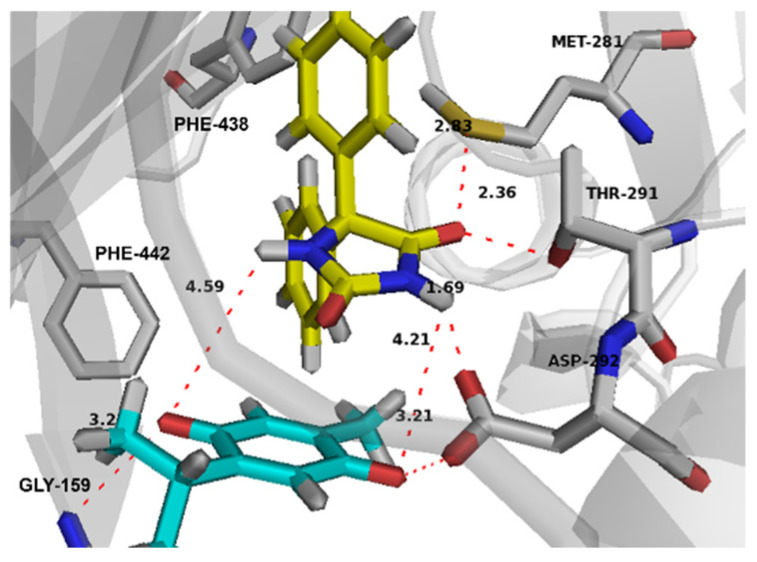
Binding interactions of co-bound PHT (carbons in yellow) and THQ (carbons in green) with the Akt active site. Distances are represented as red dotted lines and are measured in Angstrom. PHT and THQ indicate phenytoin and thymoquinone.

Both drugs cooperatively bind the active site and established a good network of intermolecular interactions (Figure 10). ASP 292 was found to be a crucial residue by simultaneously binding both drugs in the active site through strong hydrogen bonding with its carboxylate side chain and the imidazole nitrogen of PHT and the carbonyl group of THQ. Other residues that are involved in potential hydrogen bonding include MET 281 and THR 291 for PHT, and GLY 159 for THQ. The interesting poses that both drugs showed in the active site suggest that this co-binding could be further driven by a supramolecular interaction between the two drugs that makes their co-binding more energetically favored. This was noted from the π-π stacking of the PHT imidazole ring and the THQ quinone ring. There are also potential hydrogen bonds between the two forementioned rings (Figure 10).

## 3. Discussion

The goal of treating epileptic patients with current anti-epileptic drug (AED) regimens is to provide symptomatic relief and to improve the quality of life. Since 1993, 15 new AEDs have entered the market. Though the mechanism of action of some drugs are not identified yet, AEDs are usually classified according to their mechanism of action [27]. The most important mechanisms are: 1—significant reduction of continuous neuronal stimulation by inhibition of voltage-dependent sodium channel; 2—intensification of y-aminobutyric acid (GABA) inhibition; 3—inhibition of the excitatory effect of glutamate. The AEDs like vigabatrin (VIG), tiagabine (TGB), benzodiazepine (BZD) and phenobarbital (PB) act by increasing the level of GABA, either by inhibition of its reuptake or by increasing its release to the synaptic cleft [7,28]. In addition, AED combinations, like ethosuximide (ETX) with valproic acid (VPA), are reported to be more effective than the monotherapy in children suffering from absence seizures. Other AED combination regimens include lamotrigine (LTG)-levetiracetam (LEV) and lacosamide (LCM)-LEV, VPA-LTG [29]. PHT-PB (phenobarbital), carbamazepine (CBZ)-VPA, vigabatrin (VIG)-LTG, tiagabin (TGB)-VIG, gabapentin (GBP)-LTG and LTG-TPM (topiramate) [30,31,32]. However, 35% of still patients remain poor responders [33], which clearly indicates that additional mechanisms contribute to hyperexcitability and epileptogenesis. The gap in the therapeutic treatment of epilepsy prompted us to search for novel drug combinations targeting novel pathways. We tested the potential of phenytoin (PHT), thymoquonine (THQ) and their low dose combination to impede electroshock-induced convulsions and neuronal damage in rats, and the mechanism involved therein. This study fostered the synergistic effect of the low dose combinatorial regimen of PHT (20 mg/kg) and THQ (40 mg/kg) in mitigation of electro-convulsions in rats. The convulsions from electroshocks resulted in the upregulation of hippocampal m-TOR and proinflammatory markers, which was significantly abated with PHT (20 mg/kg) + THQ (40 mg/kg) combination, which indicates that the said combinatorial regimen has the potential to interfere with epileptogenesis (the process which converts a normal brain to epileptic). The synergistic action shown by the PHT and THQ combination on suppressing the mTOR signal was also investigated in silico by testing their inhibitory effect on the upstream signal, namely Akt and PI3K. There were slightly better affinities for Akt in the case of single compounds and the co-bound complex (the best results were obtained in the case of docking PHT first). Both drugs cooperatively bind the active site and establish a good network of intermolecular interactions, which seems to be further driven by a supramolecular interaction between the two drugs that makes their co-binding more energetically favored. The results from in vitro studies revealed that the combination of PHT + THQ (in the ratio of 1:2) increased cell viability by 242.81% compared to the toxic control. Thus, this study undoubtedly features the synergistic outcome of the PHT (20 mg/kg) + THQ (40 mg/kg) combination to contain seizures, interfere with epileptogenesis and provide neuroprotection.

Phenytoin (PHT) is a glycolylurea compound used in the treatment of partial seizures or tonic-colonic (grand mal), as well as acute treatment of generalized status epileptics. PHT exhibits significant antiepileptic effect by escalating the seizure threshold and reducing seizure duration [34]. It is enumerated on the World Health Organizations List of Essential Medicines. However, it has a narrow therapeutic window and overdose or chronic consumption leads to toxicity of the nervous system (insignificant nystagmus to ataxia, slurred speaking, fatigue, and ultimately stupor or death) and cardiovascular system (dysrhythmias and SA and AV nodal blocks) [35]. Thymoquinone (THQ) is extracted from the medicinal plant *Nigella sativa* (Ranunculaceae Family), famous by black seed, nutmeg flower or fennel flower [36]. It possesses pharmacological activity against oxidative stress, inflammation, and against cancer as well [37]. THQ (i.c.v. injection) increased the latency to tonic-clonic seizures in the PTZ model [38]. THQ reduced spike wave incidence and severity of epileptic activity in penicillin-provoked seizures [39]. THQ reduced seizure severity by rising Nrf2, HO-1 proteins and SOD in the Li-pilocapine model [40]. THQ increased the potency of sodium valproate (SVP) against MES and PTZ-provoked convulsions [41]. Given the therapeutic profile of PHT, we sought to combine it with THQ, a neuroprotective agent, with the motive of reducing the adverse effects of PHT, while potentiating its anticonvulsant efficacy. We succeeded as the small dose regimen of PHT (20 mg/kg) with THQ (40 mg/kg) showed synergism in alleviation of electroshock-induced seizures in rats (superior efficacy than monotherapy with PHT and THQ).

mTOR (mammalian target of rapamycin) pathway play a vital role in hereditary and acquired epilepsy states [42]. Neuronal mTOR overactivity is related to changes in seizure severity and related neuropathology in the tuberous sclerosis complex (TSC) and focal cortical dysplasia (FCD) [43]. mTOR overactivation within the hippocampus is reported in the preclinical models of temporal lobe epilepsy [44]. Rapamycin (mTOR inhibitor) reduced the seizure-induced rise of mTOR levels in rat brain tissues and reduced neuronal cell loss and mossy-fiber sprouting in pilocarpine and kainic (KA)-acid models of temporal lobe epilepsy (TLE) [45,46]. Our results endorse the previous findings, as we report hyperactivation of the m-TOR pathway upon electroshock-induced convulsions in rats (which mimics the generalized type of seizures). We report the inhibition of the m-TOR pathway with PHT at 40 mg/kg i.e. at a higher dose, while dose dependent inhibition of the mTOR pathway was detected with THQ at 20 mg/kg and 40 mg/kg. However, the synergistic low dose combination of PHT (20 mg/kg) and THQ (40 mg/kg) markedly reduced (*p* < 0.001) m-TOR activation, which indicates that the claimed synergistic combination restricts seizures and epileptogenesis by inhibiting the m-TOR pathway in epileptic rats, a novel pathway.

Cytokines include transforming growth factors (TGF), interferons, tumor necrosis factors (TNF), interleukins, lymphokines and chemokines. These are produced by the central nervous system cells (astrocytes, microglia and neurons). The concentrations of cytokine are meagre in the normal brain. However, the pro-convulsive insults upregulate the expression of cytokine and their receptors, culminating in neuronal hyper-excitation [47]. The expression of IL-1β mRNA in the cortex, hippocampus and hypothalamus increase rapidly following proconvulsive insults in experimental animal models. Overproduction of IL-1β reduces GABA-mediated neurotransmission, upregulates NMDA receptor function, and inhibits K^+^ efflux leading to neuronal hyper-excitability and convulsions [48]. Additionally, IL-6 expression is upregulated in several neurodegenerative diseases, CNS infections and injury. Studies have shown that IL-6 contributes to seizure predisposition, occurrence and seizure-related neuronal injury [49]. IL-6 mRNA and IL-6 protein in glial cells was found elevated in animal models of status epilepticus (SE), and the rapid increase of IL-6 expression is associated with membrane depolarizations [48]. Moreover, TNF-α upregulate the expression of glutaminase and gap junctions in microglia which stimulates microglial glutamate release, upregulates glutamate receptors and induces GABA receptor endocytosis, leading to proconvulsive effects [24,50]. TNF-α-administered rats exhibit prolonged seizures [24]. In a mouse model of limbic epilepsy, activation of TNF-α signaling leads to overexcitability and acute seizures following infection with Theiler’s murine encephalomyelitis virus (TMEV) [51]. The anti-inflammatory effects of THQ are reported in the literature via multiple mechanisms such as suppression of matrix metalloproteinase (MMP)-13, tumor necrosis factor alpha (TNF-a), Cox-2, IL-13, IL-5, IL-4 and prostaglandin E2 [52]. THQ also prevents nitric oxide production, a mediator of proinflammatory cytokines [53]. In this study we found that the level of hippocampal IL-1β, IL-6 and TNF-α were significantly (*p* < 0.001) higher in the toxic control group, compared to the normal control, which was reversed by PHT (20 and 40 mg/kg) and THQ (40 and 80 mg/kg) alone. THQ exhibited a higher anti-inflammatory effect than PHT. Interestingly, the most significant effect (*p* < 0.001) was observed with the synergistic low dose combination regimen of PHT (20 mg/kg) and THQ (40 mg/kg) against all cytokines (IL-1β, IL-6 and TNF-α).

Intrahippocampal kainate is reported to produce a substantial reduction of neurons in hilar regions, CA1 and CA3, while THQ drastically diminished these changes [54]. THQ restricted hippocampal morphologic derangement after prolonged toluene exposure in rats [55]. Although PHT-related polyneuropathies are rare, they are associated with large dose or plasma levels of PHT, mainly achieved throughout chronic treatment [56]. PHT prevented rat brain impairment provoked by blocking the middle cerebral artery [57]. In agreement with previous evidence, we report neuronal degeneration of hippocampal regions following electroshock-induced convulsions in rats. The THQ exhibited more neuroprotection, compared to PHT. However, the neuroprotective effects of the synergistic combination, PHT (20 mg/kg) + THQ (40 mg/kg), clearly surpass those of monotherapies (as evidenced by meagre pyknosis and grossly protected neuronal morphology).

Al-hebshi et al., 2013, reported that THQ significantly attenuated Aβ1-42-induced neuronal toxicity in rat main neurons (as proven by improved cell viability) [58]. In primary mesencephalic culture, THQ protected dopaminergic neurons by inhibiting mitochondria-mediated apoptotic cell death [59]. THQ also protected against ethanol-induced neuronal death. THQ increased Bcl-2 expression and repressed the triggering of caspase-3 and 9, and reduced the cleavage of PARP-1 [60]. In agreement with previous studies, we report an increase in cell viability following THQ treatment. Nevertheless, the synergistic combination of PHT (20 mg/kg) and THQ (40 mg/kg) improved cell viability by 242.81%, compared to the toxic control, i.e., PTZ-treated cells. The results from cell line studies validate our claims.

## 4. Materials and Methods

### 4.1. Animals

The albino wistar rats (weight range of 180–200 g) were obtained from the Central Animal House Facility, IRMC, Imam Abdulrahman bin Faisal University, Dammam, KSA. The animals were grouped randomly and housed in standard environmental conditions; 12 h light on/off cycle and precise temperature (25 ± 2 °C) and humidity (55–65%). They were fed on a regular commercial diet and water *ad libitum*. All experiments were commenced under approval no: IRB-2021-05-126 from the Institutional Animal Care and Use Committee (IACUC), IAU, Saudi Arabia.

### 4.2. Drugs and Dosing Plan

The test drugs utilized for this study were: phenytoin (PHT; 20 and 40 mg/kg), thymoquinone (THQ; 40 and 80 mg/kg). All experimental drugs were dissolved in a 2% aqueous solution of Tween 20 and administered through an oral feeding needle for 14 days before challenge with MES. The rationality of test dose selection was obtained from literature-based evidences. PHT (40 mg/kg) had been found to be protective against seizures inflicted by the 6 Hz and MES models [61], while THQ (40 and 80 mg/kg) lengthened the seizure onset against PTZ-inflicted convulsions [62].

### 4.3. Experimental Groups

The test rats were divided into seven groups of ten rats each. Group-I (normal control group) was given 10 mL/kg of vehicle. Group-II (toxic control group) was given 10 mL/kg of vehicle and later challenged with MES. Groups (Group-III–Group-VI) were administered PHT (20 and 40 mg/kg) and THQ (40 and 80 mg/kg) at two dose levels, followed by a challenge with MES. The last group (Group VII) received the low dose combination regimen of PHT (20 mg/kg) and THQ (40 mg/kg). All doses were given prophylactically for 14 days before the MES challenge.

### 4.4. MES-Induced Tonic Hind Limb Extension (THLE)

MES apparatus was used to deliver electric current to the rat via auricular electrodes. At the start of the experiment, the rats were held by hand, tethered with auricular electrodes and set free once the stimulus was enacted to observe their seizure behavior during the course of time [25]. The seizure threshold was calculated by the electric shock strength (in mA) that is required to produce THLE in 100% of normal mice (referred hereto as CS 100) [25]. The MES model was standardized at 180 mA, 220 V for 0.20 s to induce THLE in 100% rats. The MES convulsions were recorded in five stages; stage-1: tonic limb flexion, stage-2: tonic limb extension, stage-3: clonic convulsions, stage-4: stupor, and stage-5: recovery or death [63]. The test drugs were expected to possess anticonvulsant effects if they abolished THLE.

### 4.5. Experimental Design

**Figure 11:**
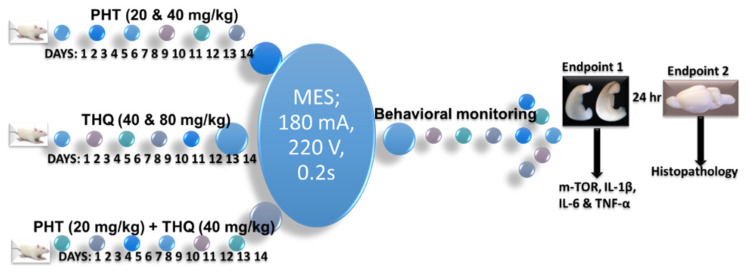
Scheme of the treatment.

The test drug (s) were administered orally once per day for 14 days prior to MES challenge (180 mA, 220 V for 0.20 s). The behavioral monitoring of convulsions was continued until the rat regained posture and/or started moving normally. Six rats were immediately sacrificed by use of sevoflurane and the brains were quickly removed on a petri dish placed over ice. The hippocampus was dissected (right and left lobe separately), washed to remove excess blood, weighed and kept stored at −80 °C in a deep freezer, until further processing. The preserved tissues were then processed for quantification of IL-1β, IL-6, TNF-α, and m-TOR levels with ELISA. The remaining four rats from each of the groups were monitored for morbidity and mortality for one day, thereafter ethically euthanized for histopathological studies (Figure 11).

### 4.6. MES-Induced Neuronal Damage

Neuronal damage incited by MES (180 mA, 220 V for 0.20 s) was accessed by histopathological assessment of the hippocampus (1 day after MES challenge). After confirming the death of the rats from the euthanasia, the brain was removed, rinsed with 0.9% saline and placed in 10% formalin. In order to assess granular cell changes, rat brains were cut coronally (8-μm thick) by microtome at the 2.3 to 4.3 mm back area to the bregma. Then segments were put on glass slides and collections of two glass slides were stained with hematoxylin and eosin. The photos for hippocampal regions, CA1, CA2, CA3, and DG, were taken by a specialist unaware of the drug treatment (at 40× for all subregions, using a light microscope) [25].

### 4.7. Assessment of Hippocampal m-TOR Levels

After recovery of rats from electroshock-induced convulsions, they were sacrificed and the brains were taken out, the hippocampus extracted and processed for the estimation of mTOR. Immunoassay using the protocol-based kit from Aviva Systems Biology (San Diego, CA, USA) was conducted to quantify the mTOR levels in the hippocampus region.

### 4.8. Assessment of Proinflammatory Markers in the Hippocampus

After recovery of rats from electroshock-induced convulsions, they were sacrificed, the brains were immediately removed and the hippocampus tissues were extracted and preserved in freezing phosphate buffer (pH: 7.4). After that, hippocampus tissues were chopped and centrifuged at 5000 rpm at 4 °C for 15 min to obtain the supernatant, which was collected in a separate tube. The *IL*-1β, IL-6, and TNF-α levels in the supernatant were measured using the immunoassay protocol-based kit from Aviva Systems Biology (San Diego, CA, USA).

### 4.9. In Vitro Studies

#### 4.9.1. Cell Culture

The human embryonic kidney cells (HEK-293) were utilized to examine the effect of test drugs and their combinations on cell viability. The HEK-293 cells are widely used in neuroscience-related studies, as several neuron-specific genes are found in HEK293 cells [64,65]. The HEK-293 were cultivated in 96-well plates containing DMEM media supplemented with fetal bovine serum, L-glutamine, selenium chloride, penicillin and streptomycin as per the methods described [66,67,68]. The cells were kept for at least 48 h in the (5%) CO_2_ incubator at 37 °C (as we needed 70–80% confluence cells).

#### 4.9.2. In Vitro Model of Cellular Degeneration

We replicated cellular degeneration by treating HEK-293 cells with pentylenetetrazol (PTZ; 0.6 µg/mL) for 24 h, after which the cellular morphology and anatomy was observed under a light microscope. In the control group, test drug/s were not added. The PTZ is used to model absence seizures of generalized type and kindling seizures in experimental animals [69,70,71].

#### 4.9.3. Treatment with Test Drugs

After treatment with PTZ (for 24 h), the HEK-293 cells were treated with test drugs and their combinations: PHT (0.33 µg/mL), THQ (0.33 µg/mL) and PHT (0.33 µg/mL) + THQ (0.66 µg/mL). The dose of this combination, PHT and THQ at a ratio of 1:2, was based on the doses administered to rats. We used different concentration of drugs ranging from 0.2–0.90 µg/mL.

#### 4.9.4. MTT Assay

After 24 h of treatments, cells were incubated with MTT (5.0 mg/mL) and were preserved in incubation for 4 h. The cells were then washed, and 100 µL DMSO was poured in each well. The percentage of cell viability was measured by an ELISA Plate Reader at 570 nm (Biotek Instruments, Winooski, VT, USA).

### 4.10. Molecular Modeling

MOE was utilized for molecular docking simulation and ligand binding energy calculation. Pymol software was used for data visualization output and figure generation. The docking studies utilized crystalline structures of human Akt (PDB code; 4gv1), and PI3K (PDB code; 1e7v) co-crystallized with inhibitors. After deleting the co-crystallized inhibitors, all hydrogens were added to the ligand PDB file and partial charges were computed. The amino acid residues involved in binding the co-crystallized ligand was used to define the active site for ligand binding.

### 4.11. Statistical Analysis

ANOVA preceded by post hoc Dunnet’s test was used to detect the difference in mean of the toxic control vs. other groups. In order to determine the ratio of THLE/NO THLE, Fischer’s exact test (one tailed) was utilized. * *p* < 0.05, ** *p* < 0.01 and *** *p* < 0.001 were considered significant, highly significant and extremely significant in all cases, respectively. GraphPad software was used for statistical analysis.

## 5. Conclusions

Epilepsy is a global health concern. Use of anti-epileptic drug is advisable at certain stages with acceptable side effects, but 35% of patients remain refractory even with multiple AED regimens, which makes it important to search for novel chemical moieties or combine existing drugs with the motive of achieving better efficacy and reduction of adverse effects. Therefore, we sought to combine PHT, an anticonvulsant drug, with THQ, a neuroprotective agent, to enhance the anti-convulsant efficacy of PHT and interfere with the process of epileptogenesis (i.e., exhibit neuroprotection). Fortunately, we succeeded, as the combination (PHT + THQ) regimen exhibited synergism in abrogation of convulsions, reduced neuroinflammation and downregulated the m-TOR pathway (which is hyperactivated during seizures and contributes to epileptogenesis). The low dose of this combination regimen also increased cell viability in in vitro assays. This combination thus seems to have the possibility of treating generalized seizures and refractory cases.

## Figures and Tables

**Table 1:** Dockings scores for PHT, THQ alone and co-bound in Akt and PI3K.

	Docking Score			
	PHT 1st	PHT/THQ	THQ 1st	THQ/PHT
Akt	−10.1	−9.1	−6.4	−5.6
PI3K	−9.4	−9.2	−5.9	−6.5

## Data Availability

Data is contained within the article.
